# Frequency of Blood Types A, B and AB in a Population of Non-Pedigree Domestic Cats from Central Italy

**DOI:** 10.3390/ani10101937

**Published:** 2020-10-21

**Authors:** Morena Di Tommaso, Arianna Miglio, Paolo Emidio Crisi, Andrea Boari, Francesca Rocconi, Maria Teresa Antognoni, Alessia Luciani

**Affiliations:** 1 Faculty of Veterinary Medicine, Veterinary University Hospital, University of Teramo, Piano d’Accio, 64100 Teramo, Italy; pecrisi@unite.it (P.E.C.); aboari@unite.it (A.B.); frocconi@unite.it (F.R.); aluciani@unite.it (A.L.); 2 Department of Veterinary Medicine, University of Perugia, 06100 Perugia, Italy; maria.antognoni@unipg.it

**Keywords:** feline, AB blood group, transfusion reaction, neonatal isoerythrolysis, central Italy

## Abstract

**Simple Summary:**

This study aimed to evaluate the frequency of AB blood groups in non-pedigree domestic cats in Central Italy. The presence of natural alloantibodies in the feline blood group system can cause life-threatening transfusion reactions due to mismatched transfusion and, depending on the parents’ blood type, can also account for hemolytic reactions in newborn kittens. Based on the frequency of the AB blood groups found in the 483 cats studied, a potential risk (6.5%) of acute hemolytic transfusion or neonatal reactions was calculated. Therefore, determining the blood group in non-pedigree domestic cats from Central Italy before transfusion is strongly recommended, as has been reported for other geographical areas. Moreover, particular attention should be paid to the mating of non-purebred cats.

**Abstract:**

Blood transfusion reactions and neonatal isoerythrolysis are common events in the feline population due to the presence of natural alloantibodies in the AB blood group system. It is known that the frequency of feline blood types varies according to the geographic region and breed. Therefore, the aims of this study were to investigate the frequency of AB blood groups in non-pedigree domestic cats in Central Italy and estimate the risk of a life-threatening transfusion reaction and neonatal isoerythrolysis, caused by mismatched transfusion or incompatible random mating, respectively. The AB blood group was determined on non-pedigree domestic feline patients and potential blood donors submitted at the Veterinary Teaching Hospitals of the Universities of Teramo (Abruzzo Region, Teramo, Italy) and Perugia (Umbria Region, Teramo, Italy), and visited at veterinary practitioners in Rome (Lazio Region, Teramo, Italy) using commercial immunochromatographic cartridges and commercial agglutination cards. There were four hundred and eighty-three cats included in the study. The frequencies of the blood types were: 89.9% type A, 7.0% type B, and 3.1% type AB. The probability of an acute hemolytic transfusion reaction or a neonatal isoerythrolysis was 6.5%. Although the frequency of type B in non-pedigree domestic cats living in Central Italy was relatively low, to reduce the risk of fatal transfusion reactions, blood group typing is recommended before each transfusion.

## 1. Introduction

The AB blood group system is the main feline erythrocytes antigen system that includes blood types A, B, and the rare AB. Determining blood types is of clinical importance as type A and type B cats possess naturally occurring alloantibodies against the blood type antigen they lack and type AB cats have no anti-AB alloantibodies. Particularly, type B cats generally have high titers of anti-A isoagglutinins, therefore, a transfusion of type A or type AB blood to a type B recipient or incompatible mating can cause, respectively, very serious acute hemolytic transfusion reactions and neonatal isoerythrolysis (NI) in type A and type AB kittens born from a type B mother [1].

The frequency of blood types in cats varies geographically and within breeds. Several international studies show that type A is the most common blood type in non-pedigree cat populations in different European countries [2,3,4,5,6,7,8,9,10,11,12,13,14,15,16,17,18,19,20,21,22,23,24,25]. On the other hand, it has been seen that Type B and the rare type AB has a high incidence in some feline pure breeds and no incidence in other breeds [8,26,27,28].

Studies to determine the frequency of blood groups in domestic cats in Italy produced similar results to those reported in the international literature, with type A overrepresented in the feline population. However, these studies describe mainly feline populations from Northern Italy, whereas data on blood types frequency in cats from Central and Southern Italy are limited [5,13,14,15,16].

Therefore, the study aims to firstly investigate the frequency of AB system blood groups in a non-pedigree domestic cats population of Central Italy (including animals from Abruzzo, Umbria, and Lazio Regions), and secondly, to estimate the risk of a life-threatening transfusion reaction or NI caused by a mismatched transfusion or incompatible random mating, respectively.

## 2. Materials and Methods

Between January 2016 and October 2019, 483 non-pedigree domestic cats were admitted to the Veterinary Teaching Hospitals of the Faculty of Veterinary Medicine of Teramo, Abruzzo Region, Italy (VTHT) and of the Department of Veterinary Medicine Perugia, Umbria Region, Italy, (VTHP) and to veterinary practitioners (VP) in Rome, Lazio Region, Italy as potential blood donors following the national guidelines for transfusion medicine [28,29,30,31], or for diagnosing different diseases or vaccinations were included in the survey.

Blood samples from each cat were obtained by jugular venipuncture and collected into EDTA tubes (1 to 2 mL). Blood typing was immediately performed using commercial immunochromatographic cartridges (IC—LabTEST or QuickTEST A+B, Alvedia, Lyon, France) or commercial agglutination cards (AC—RapidVet-H Feline, Agrolabo, Torino, Italy). All subjects from the VTHP and the VP were typed by IC. Type B and AB results from VTHP and VP were back-typed as previously described [14,16] or retyped by IC, respectively, to be confirmed. All potential donors and all anemic cats from VTHT were typed and retyped using IC, whereas all the B and the AB patients that had been previously tested by AC were retyped by IC. Ethical review and approval were not required for this study as all the animals admitted to the VTHs and VP, with consent from the owners, routinely undergo blood withdrawal and typing. Written informed consent was obtained from the owners for blood sampling, analysis, and to allow their animals to participate in this study.

The percentage of each blood type was determined. The χ^2^ test was used to evaluate the association between the frequency of blood type and gender. A *p* value < 0.05 was considered significant.

The potential risks of developing an acute hemolytic reaction caused by a transfusion from a type A or type AB donor to a type B recipient, and of developing NI in type A or type AB kittens born from the mating between a type B queen and type A or type AB tom, were calculated by multiplying the percentage of type B cats by the percentage of non-type B cats (type A + type AB) [32].

## 3. Results

Of the 145 cats initially included in the study from VTHT, 10 samples that had been tested by AC were excluded; 6 showed autoagglutination in the control well and 4 a hematocrit <10%, and retyping by IC was not possible because the available blood was not sufficient to perform additional tests.

All the B and the AB cats from VTHT and VP that had been typed and retyped by IC showed an obvious positive reaction to the B antigen and the control.

All the B and the AB results from VTHP were confirmed by back-typing.

The samples initially tested using AC were 29. Of the 29 samples tested by AC, 1 was type AB, 2 type B and 26 type A.

Subjects enrolled were all mixed breed cats, 58% males and 42% females, with a median age of 3 years (range, 0.1–17 years).

The frequencies of the blood types in the feline population tested were as follows: 89.9% type A, 7.0% type B, and 3.1% type AB. All cats (*n*. 252) from VTHP were potential donors, whereas cats from VTHT (*n*. 135) and VP (*n*. 96) were potential donors, and patients. Of the 127 patients, all FeLV negative, type B cats (*n*. 13) were represented by five anemic patients with chronic kidney disease (Hct between 18% and 26%), four cases of squamous cell carcinoma, and one subject for each of the following diseases: feline non-flea hypersensitivity dermatitis, hind limb fracture, otoacariasis, and hypertrophic cardiomyopathy. Type AB cats were represented by 1 cholangiohepatitis, 1 jaw fracture, 1 diaphragmatic hernia, and 1 feline scabies.

There was no statistical association between the frequency of blood type and gender (*p* = 0.37).

The risk of an acute hemolytic transfusion reaction or a NI was 6.5%.

## 4. Discussion

The present study shows that the non-pedigree domestic feline population in Central Italy is predominantly of blood type A (86.7%), as reported in many other European and Italian geographic areas (Table 1). The reason for this is that the allele *A* is dominant to the *b* allele, and the *a^ab^* allele is dominant to b and recessive to the A allele, resulting with the following genotypes/phenotypes: *AA*, *Ab* and *Aa^ab^* for type A, *b/b* for type B, and *a^ab^b* and *a^ab^a^ab^* for type AB [33].

As reported in Table 1, in this survey the frequency of type B in the feline population is higher (7.0%) compared to most other European studies (i.e., Croatia, Denmark, Finland, Hungary, Iberian Peninsula, Switzerland, and UK) [2,3,5,10,11,17,18,19,21] and is comparable to those of four studies conducted in Northern Italy, where about a total of 600 European domestic shorthair cats were enrolled [13,14,15,16]. Instead, Giger et al. and Spada et al. reported a high prevalence of blood type B in Central (11.2% in Tuscany region) and Southern Italy (12.1% in Sicily), respectively [5,16]. Although in most reports from other European areas the prevalence of group B in non-pedigree cats is also low (less than 10%), there are marked geographical variations. Previous studies present a variability of type B prevalence ranging from 0% in Croatia, Finland and Hungary to 30.5% in England and over 20% in other countries (i.e., Turkey, Germany, and Greece) [2,4,5,8,9,10,22,23].

In this study, the blood type AB was quite low (3.1%) and this result is in contrast with the high incidence identified in Southern Italy in a recent study [16]. To the author’s knowledge, this result is similar to that reported in most other studies with the exception of Portugal, Germany, and UK where a frequency of 6.3%, 7.4%, and 8.5% in mixed breed cats was reported, respectively [8,17,25]. Furthermore, the results reported in the present study are similar to those blood types found in feline populations tested in other areas of Italy, with the exception of type AB group [13,14,15,16].

Knowledge of the prevalence of blood type in cats is important to prevent both serious transfusion reactions and NI. While type A cats have low titers of naturally occurring anti-B isoagglutinins (between 1:4 and 1:32) in only one-third of subjects, which cause premature destruction of eventually transfused type B red blood cells, unmatched blood transfusion of type A to a recipient with blood type B, even only 1 mL, causes a life-threatening acute hemolytic reaction [34]. This is because type B cats have high titers of anti-A alloantibody mainly of the IgM class (between 1:2 and 1:1600, generally >1:64) with haemagglutinating and haemolysing activity [35,36]. For the same reasons, blood type A or AB kittens born to a type B queen can develop a form of potentially fatal hemolytic anemia in the first hours of life after the gastrointestinal absorption of anti-A antibodies present in the colostrum [37]. Based on our results on frequencies of blood types A, B, and AB in the feline population examined, the potential risks of life-threatening immune-mediate transfusion reactions or NI were 6.5%. This means that about one out of fifteen cats receiving an unmatched transfusion is at risk of developing fatal hemolytic anemia. Therefore, although the potential risks of developing an acute hemolytic reaction are not particularly high, blood typing of all donors and patients is the first step to ensure safe and compatible transfusion in all mixed breed cats in Central Italy.

At the same time, although NI is an important disease in purebred cats, a random mating of crossbreed cats could result in the death of kittens by NI with an equally unacceptable probability. However, it is true that the development of this disease also depends on other factors such as the quantity and quality of the colostral antibodies uptake by the kittens. Therefore, not all kittens are at risk of developing the same degree of hemolysis or the severity of clinical manifestations [38].

It is worth mentioning that the transfusion outcome is also related to the presence of naturally occurring non-AB antibodies against a recently discovered and named *Mik* feline antigen. The presence of anti-*Mik* alloantibodies in *Mik*-negative recipient cats can potentially cause an acute hemolytic anemia post *Mik*-positive blood transfusion [39]. A recent investigation has shown a prevalence of 15% of non-AB blood type incompatibilities in 154 transfusion-naive domestic cats, likely related to the presence of naturally occurring *Mik*-alloantibodies [40]. Therefore, it is recommended to always perform a pre-transfusion crossmatch in all feline blood transfusion cases, regardless of blood type and transfusion history.

The lack of back-typing of B and AB samples from the VTHT and the VP (*n*. 20 and *n*. 6, respectively) could be a limitation of this work. However, the immunochromatographic test has proven to be a method with high sensitivity and specificity in detecting feline blood types A, B, and AB [41]. In all 231 samples tested by IC in the VTHT and in the VP, the retyping with IC confirmed the result of the first typing. In addition, the retyping also confirmed the positivity in the control band, therefore theoretically, any potential autoagglutination should not have interfered with the results of the immunochromatographic test and would not misclassified type B and AB cats.

## 5. Conclusions

This study shows a relatively low prevalence of blood type B in non-pedigree domestic cats living in Central Italy (Abruzzo, Umbria, and Lazio regions). However, to reduce the probability of fatal transfusion reactions, blood group typing of donor and recipient cats and crossmatch must always be performed before all blood transfusion, even in low-risk populations.

## Figures and Tables

**Table 1:** Frequencies of the AB system blood types in non-pedigree cats in various European countries including this study.

Country (Date)	Number	Blood Type (%)		
		A	B	AB
Present study	483	89.9	7.0	3.1
Croatia (2016) [2]	30	96.7	0.0	3.3
Denmark (1994) [3]	105	98.1	1.9	0.0
England (2007) [4]	105	67.6	30.5	1.9
Finland (1992) [5]	61	100.0	0.0	0.0
France (2017) [6]	231	89.6	10.0	0.4
France (2019) [7]	320	83.7	14.4	1.9
Germany (2018) [8]	81	71.6	21.0	7.4
Greece (2001) [9]	207	78.3	20.3	1.4
Hungary (2001) [10]	81	100.0	0.0	0.0
Iberian Peninsula (2017) [11]	827	96.9	3.1	0.0
Ireland (2011) [12]	137	84.7	14.6	0.7
Italy (1992) [5]	401	88.8	11.2	0.0
Italy (2007) [13]	122	86.9	7.4	5.7
Italy (2011) [14]	140	90.7	7.1	2.1
Italy (2014) [15]	195	92.3	5.1	2.6
Italy (2020) [16]	448	84.4	8.5	7.1
Portugal (2004) [17]	159	89.3	4.4	6.3
Portugal (2011) [18]	515	97.5	2.1	0.4
Spain (2004) [19]	100	94.0	5.0	1.0
Spain (2004) [20]	97	88.7	7.2	4.1
Switzerland (1993) [21]	1014	99.6	0.4	0.0
Turkey (2006) [22]	301	73.1	24.6	2.3
Turkey (2010) [23]	240	72.1	25.4	2.5
UK (1999) [24]	139	87.1	7.9	5.0
UK (2014) [25]	82	79.3	12.2	8.5
